# 

**Published:** 1891-07

**Authors:** 


VOL. XII.] *PUBLISHED MONTHLY AT THREE DOLLARS A YEAR. SINGLE COPIES, THIRTY CENTS.* [No. 7.

LIBRARY Indexed S. G. O.

### THE JOURNAL OF COMPARATIVE MEDICINE AND VETERINARY ARCHIVES.

EDITED BY
W. A. CONKLIN, Ph. D., D. V. S.,
*Director of Zoological Gardens, New York.*
RUSH SHIPPEN HUIDEKOPER, M. D., Veterinarian, (*Alfort.*)
*Professor of Sanitary Medicine and Veterinary Jurisprudence, American Veterinary College, New York.*

JULY, 1891.

All communications and payments should be addressed to

**MANAGING EDITOR JOURNAL COMP. MED. AND VET. ARCH.**

FORTY-SECOND STREET AND PARK AVENUE, NEW YORK.

Printed for the Editors by

WILLIAM R. JENKINS, PUBLISHER,
NEW YORK.

**LONDON: BALLIERE, TINDALL & COX.**

